# Optimistic Environmental Messaging Increases State Optimism and *in vivo* Pro-environmental Behavior

**DOI:** 10.3389/fpsyg.2022.856063

**Published:** 2022-04-29

**Authors:** Megan MacKinnon, Adam C. Davis, Steven Arnocky

**Affiliations:** Human Evolution Laboratory, Department of Psychology, Nipissing University, North Bay, ON, Canada

**Keywords:** pro-environmental behavior, pessimism, optimism, priming experiment, conservation behavior, *in vivo* environmental action

## Abstract

Despite recent empirical interest, the links between optimism and pessimism with pro-environmental behavior (PEB) remain equivocal. This research is characterized by a reliance on cross-sectional data, a focus on trait-level at the neglect of state-level optimism–pessimism, and assessments of retrospective self-reported ecological behavior that are subject to response bias. To attend to these gaps, 140 North American adults (*M*_age_ = 34; *SD* = 11.60; 44% female) were experimentally primed with bogus optimistic or pessimistic environmental news articles, and then asked to report their levels of state optimism–pessimism, intentions to purchase green products, *in vivo* PEB (donating to WWF and providing contact information to join an environmental organization), and support for geoengineering technologies. Results confirmed that optimistic (versus pessimistic) environmental messaging enhanced the expression of state optimism, which then contributed to PEB and support for geoengineering. These results have important implications for the framing of environmental messaging intended to promote ecologically conscious behavior.

## Introduction

Many people across the globe are concerned about the threat posed by climate change, which may evoke feelings of uncertainty, anxiety, and hopelessness (Landry et al., 2018; Clayton, 2020). Expressing concern and feeling hopeful about the future may motivate pro-environmental behavior (PEB) and engender trust that one’s actions can ameliorate pressing ecological dilemmas (Ojala, 2012, 2015). Conversely, hope may be underpinned by “wishful thinking” and denialism, leading to complacency and inaction (Ojala, 2012). Furthermore, negative emotionality (e.g., fear) may be important in being able to identify climate change as a threat and encourage conservation behavior (Kleres and Wettergren, 2017). Indeed, being able to acknowledge environmental dissolution and wanting to avoid negative future outcomes might motivate compensatory PEB (i.e., the constructive pessimism hypothesis; Kaida and Kaida, 2016a). Nonetheless, like the emotion of hope, heightened optimism (i.e., expressing positive future expectancies) may inspire people to take charge and engage in conservation behavior (Kaida and Kaida, 2017, 2019; McAfee et al., 2019). Accordingly, ambiguity remains surrounding the relative roles of optimism and pessimism in predicting PEB. This literature is further limited by a reliance on cross-sectional data and retrospective indices of environmental behavior. This precludes an assessment of the causal relations among variables and leaves unanswered the question of whether optimism is truly complicit in promoting conservation behavior. The objective of the current research was to address these gaps by (1) using an experimental approach by exposing participants to either optimistic or pessimistic environmental messaging, (2) examining the effects of such exposure on state optimism, and (3) examining the mediating role of state optimism induction on *in vivo* group differences in environmental attitudes and behavior. Based on recent literature reviews (McAfee et al., 2019), we anticipated that optimistic (but not pessimistic) messaging would predict a suite of *in vivo* pro-environmental attitudes and behaviors, and that induced state optimism would mediate these links.

### Optimism, Pessimism, and Environmental Action

Since the late 1960s, the proportion of negative environmental news articles in the media has increased, while the amount of positive environmental news has decreased (McAfee et al., 2019). It is important to discuss and draw attention to evidence of growing environmental degradation, but media coverage of the environment has an evident negativity bias: coverage of environmental threats and the failure of conservation efforts receives significant attention, whereas ecological recovery and successful conservation is largely ignored (Ader, 1995; Hart and Feldman, 2014; McAfee et al., 2019). Despite the prevalence of environmental messaging in the media, there is limited empirical work on the topic of how pessimistic and optimistic environmental messaging may influence viewers’ attitudes, values, and behavior toward the environment (Morris et al., 2020). There is also a shortage of research considering individual differences in optimism and pessimism in relation to PEB (Kaida and Kaida, 2019). Most of the previous research on the topic inside and outside of environmental psychology involves examining the links between related constructs, such as subjective wellbeing, in relation to PEB.

In a recent meta-analysis of 78 published studies, Zawadzki et al. (2020) found a small positive correlation between different kinds of PEB and various indicators of subjective wellbeing. In the 10% (*n* = 7) of studies that involved an experimental design, evidence suggested that positive affective states could be both an antecedent, as well as an outcome, of PEB (van der Linden, 2018). It was also uncertain whether expectations of future wellbeing and satisfaction could similarly promote, or be promoted by, PEB. In their work, Kaida and Kaida (2016a,b) found that PEB positively predicted *current* subjective wellbeing, but that *future* subjective wellbeing negatively predicted PEB. These authors reasoned that negative future expectancies might stimulate action to attenuate anxiety (so-called “constructive pessimism”), which explains why those with lower future subjective wellbeing might engage in more self-reported PEB. In contrast, Kaida and Kaida (2017) found that trait pessimism (related to lower future subjective wellbeing) shared a small negative relation with switching off the lights when not in use, whereas optimism was unrelated to this behavior. In a follow-up study, different kinds of PEB (e.g., reusing bags for grocery shopping) shared small positive relations with optimism and were either unrelated or weakly negatively related to pessimism (Kaida and Kaida, 2017). In a recent longitudinal study, Kaida and Kaida (2019) found that optimism both positively predicted and was predicted by PEB, but that the former pathway (optimism ➔ PEB) was stronger than the latter (PEB ➔ optimism). Within time, pessimism was weakly related to PEB in a negative direction. In contrast, Morris et al. (2020) found that experimentally priming pessimistic messaging about climate change increased risk perception and enhanced outcome efficacy—measured with the item “*I believe my actions have an influence on climate change*”—which was mediated by heightened emotional arousal. However, neither retrospective nor actual PEB were assessed in this study.

There is also indirect evidence supporting links between optimism-related constructs and PEB. For instance, those higher in place attachment may be happier and more optimistic (e.g., Brehm et al., 2004). Individuals high in ecological place attachment (e.g., to a national park) experience greater subjective wellbeing and are more likely to engage in PEB to protect that ecological resource (Ramkissoon et al., 2018). This research also indicates that intentions to engage in conservation behavior may be driven by certain factors encompassed within place attachment, such as place affect (e.g., feeling emotionally connected to a national park; Ramkissoon et al., 2013; Ramkissoon and Mavondo, 2014). This sense of place attachment may also be cultivated in one’s household during periods of place confinement, such as the during the COVID-19 pandemic, through engaging in household PEB (e.g., conserving water when washing one’s hands) which could heighten people’s sense of happiness and wellbeing (Ramkissoon, 2020).

Most previous research on indicators of subjective wellbeing and optimism–pessimism in relation to PEB has (1) been cross-sectional or correlational across a few longitudinal timepoints, precluding an understanding of the causal links between variables, (2) is characterized by mixed findings (e.g., Kaida and Kaida, 2016a,b, 2017, 2019), and (3) has relied on self-reported measures of retrospective conservation behavior that are subject to response bias (see Dupuis and Arnocky, 2012). To ascertain “true” causal associations and actual engagement in PEB, an experimental approach is needed with *in vivo* assessments of behavioral engagement. Previous research has explored *in vivo* measures of environmental donating and joining an environmental organization as behavioral indices of pro-environmental action, finding that learned helplessness predicted lower likelihood of engaging in these actions (Landry et al., 2018). Given that optimism is often considered in opposition to helplessness (Seligman, 2000), we anticipate that these variables will be positively associated with induced optimism. Similarly, green purchasing has previously been associated with optimism, but not pessimism (Sadiq et al., 2020). Geoengineering represents a collection of technologies intended to abate climate change that people appear to express support of and optimism toward (Rehman et al., 2021). In previous work, Landry et al. (2018) found a positive relation between support for geoengineering and environmental concern. Nonetheless, technologies designed to mitigate climate change may discourage people from engaging in everyday “green” behavior (Murtagh et al., 2015; Mittiga, 2019). It is currently unclear how attitudes toward geoengineering may be influenced by optimistic and pessimistic environmental messaging, state optimism–pessimism, and whether these attitudes may subsequently predict *in vivo* PEB. Given ambiguity surrounding how geoengineering support relates with other measures of pro-environmental attitudes and behavior (Arnocky et al., 2020), and whether optimism around the effectiveness of these technologies or pessimism about any other less risky solutions being viable (i.e., a “last resort hypothesis”) would drive support for geoengineering, we included it in an exploratory manner along with other measures of individual pro-environmental actions.

There has also been a preoccupation with trait-level (i.e., enduring) at the relative neglect of state-level (i.e., temporary) optimism–pessimism; the latter of which may be particularly important when considering the influence of environmental messaging about climate change (see Morris et al., 2020).

There is also ambiguity revolving around whether trust, hope, and optimism toward science and technology to solve environmental dilemmas detracts from (Dunlap et al., 2000), or encourages pro-environmental attitudes and action (Ojala, 2012, 2015).

## Present Study

Although challenging to advance a directional hypothesis, previous research suggests that optimistic environmental messaging likely increases state-level optimism, which may then encourage PEB (McAfee et al., 2019). The goal of the present research was to experimentally examine the effects of exposure to optimistic versus pessimistic environmental messaging upon state optimism and a diverse set of *in vivo* pro-environmental attitudes and behaviors, including green purchasing intentions, donating study earnings to an environmental organization, joining an environmental activism group, and support for geoengineering technologies. Specifically, we hypothesized:

*H1*: Exposure to optimistic (versus pessimistic) environmental messaging will predict greater state optimism.

*H2*: State optimism will predict each of the four pro-environmental outcome measures and will mediate links between condition and these outcomes.

## Materials and Methods

### Participants and Procedure

We aimed to expose participants to optimistic or pessimistic passages modified from real digital print media sources, and subsequently assess state optimism followed by measures of pro-environmental attitudes and behavior. Sample size was determined for the path analysis model based upon previous simulation studies which indicate that simple structural equation models, which path analysis falls under the umbrella of, with reliable measures, good fit indices, and limited missing data will converge with sample sizes around 100 (Iacobucci, 2010). For data that are approximately normally distributed with a small number of variables, a participant to parameter ratio of 5:1 is adequate (Bentler and Chou, 1987). With 19 parameters in the current study, a sample size of *N* = 95 would be sufficient. Previous work also indicates that to maintain adequate power (≥0.80) to detect a medium correlational effect (*r* = 0.30) *via* path analysis requires a sample size of *N* ~ 80 (Miles, 2003). Accordingly, 152 North American participants were recruited *via* Amazon’s Mechanical Turk (MTurk) and completed all tasks on Qualtrics. Participants with duplicate IP addresses, who failed to complete the experiment, failed the attention checks (“*If you are paying attention to this survey, please select ‘disagree’*”; “*What do you think this article was about?*”), or did not provide a unique survey code were removed from the analytic sample. The final sample size was 140 (*M*_age_ = 34, *SD* = 11.60, range = 19–69; 44% female). Participants were primarily Caucasian (87%), Black (10%), Latin American (2%), and South Asian (1%). Participants were renumerated $1.00 USD. This research received approval by the Nipissing University Research Ethics Board (protocol #102658).

### Measures

#### Experimental Priming Tasks

Participants were randomly assigned to one of two conditions: environmental optimism or environmental pessimism using bogus magazine articles adapted from real news (see Supplemental Material file for articles). In the optimism condition, the article explained reasons to be optimistic for the future of the environment (e.g., conservation efforts are saving many endangered species). In the pessimism condition, the article pertained to the inevitability of environmental degradation (e.g., at this point, our window for action has closed; there is nothing we can do to stop it). Participants were required to briefly explain the contents of the article that they had read as an attention check.

#### State Optimism

The 7-item State Optimism Measure (SOM; Millstein et al., 2019) was used to assess state optimism (e.g., “*The future is looking bright for me”*). Items were measured with a 5-point Likert-type scale ranging from 1 (*Strongly disagree*) to 5 (*Strongly agree*) and averaged to create a mean scale score with higher scores reflecting greater state optimism (*α* = 0.93).

#### Intended Green Purchasing

Intended green purchasing behavior was measured using the Green Purchasing Behavior Scale (Lee, 2009), which was modified slightly to address intended green purchasing behavior (e.g., “*I intend to buy organic products”*; *α* = 90). Participants responded to seven items using a 5-point Likert-type scale ranging from 1 (*Strongly disagree*) to 5 (*Strongly agree*).

#### *In vivo* Pro-environmental Behavior

Participants were given the option to keep their earnings from the survey or donate to the World Wildlife Fund (WWF), a prevalent environmental organization (coded: 0 = keep money, 1 = donate money). They were then asked if they wanted to join a bogus environmental group by providing their email (coded: 0 = no email, 1 = email provided). Donators comprised 11% (*n* = 17), and email joiners comprised 43% (*n* = 65) of the total sample. See Supplemental Material for wording of questions.

#### Geoengineering

Following Pidgeon et al. (2012), participants were asked to report their support of geoengineering defined as “*The use of large-scale engineering projects designed specifically to comebat global climate change*.” Participants used a 5-point Likert-type scale to record their responses, ranging from 1 (*Strongly oppose*) to *5* (*Strongly support*).

### Analytic Approach

**Figure 1:**
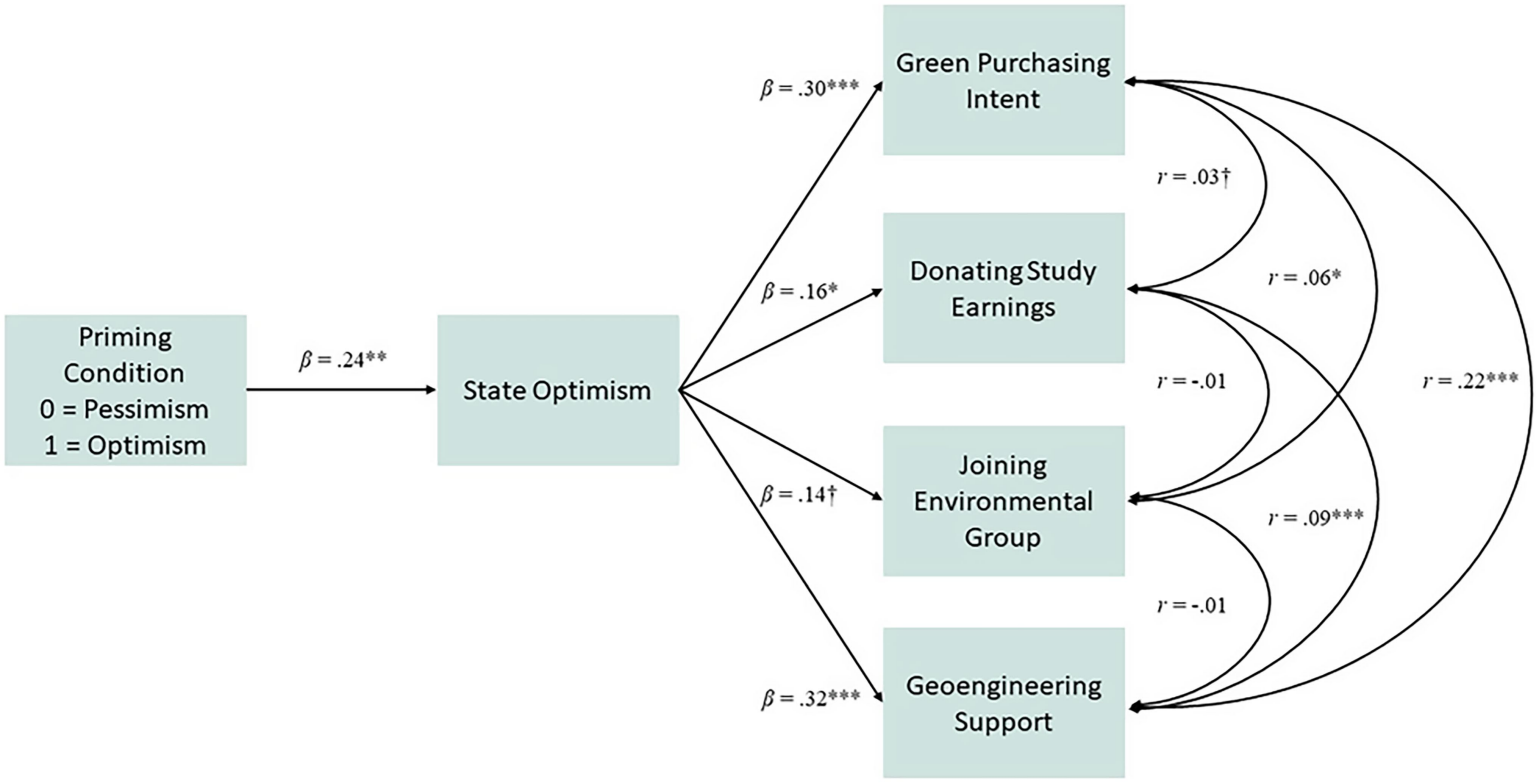
Results of an observed variable path model analysis for examining the indirect effect of induced optimism on pro-environmental behavior, green purchasing intent, and support for geoengineering. Note that links between demographic control variables sex and age and the dependent variables are not depicted. ^†^ = *p* < 0.10, ^*^ = *p* < 0.05, ^**^ = *p* < 0.01, and ^***^ = *p* < 0.001.

Path analysis *via* AMOS (version 27) was used to explore whether priming environmentally related optimism (versus pessimism) would increase state optimism (Hypothesis 1), which in turn would predict an increase in green purchasing intent, willingness to donate earnings from the study to an environmental group, and willingness to join an environmental activism group, as well as support for geoengineering (Hypothesis 2), while controlling for sex (male/female) and age (Figure 1). Path analysis allowed us to simultaneously test predictions for each dependent variable while controlling for their shared variance. Model fit was assessed using the chi-square test of significance (*χ*^2^), comparative fit index (CFI), normed fit index (NFI), and the root mean square error of approximation (RMSEA; Kline, 2016). CFI and NFI values >0.90, RMSEA values <0.08, and a non-significant *χ*^2^ indicate adequate model fit (Hooper et al., 2008). Indirect (mediation) effects were examined using 2000 bootstrap samples and bias-corrected 95% confidence intervals. Missing data were estimated using the AMOS regression imputation (Collier, 2020).

## Results

We first examined covariates and intercorrelations among dependent variables. Green purchasing correlated with joining the environmental group, *r* = 0.18, *SE* = 0.03, *p* = 0.03, and was modestly correlated with donating to the WWF, *r* = 0.15, *SE* = 0.02, *p* = 0.08. However, donating and joining the environmental group were unrelated to one another, *r* = −0.05, *SE* = 0.01, *p* = 0.56. Geoengineering support correlated positively with donating, *r* = 0.30, *SE* = 0.03, *p* < 0.001, and with green purchasing intent, *r* = 0.49, *SE* = 0.04, *p* < 0.001, but not with joining the environmental group, *r* = 0.07, *SE* = 0.02, *p* = 0.40. Sex was modestly related to green purchasing, *b* = 0.20, *β* = 0.13, *SE* = 0.13, *p* = 0.10, and joining the environmental group, *b* = 0.14, *β* = 0.14, *SE* = 0.08, *p* = 0.08, such that women were more likely than men to endorse both. However, sex was unrelated to donating, *b* = 0.01, *β* = 0.01, *SE* = 0.05, *p* = 0.93, and geoengineering support, *b* = −0.07, *β* = −0.05, *SE* = 0.10, *p* = 0.52. Younger participants were more likely to join the environmental group, *b* = −0.01, *β* = 0.26, *SE* = 0.03, *p* = 0.001, and to support geoengineering, *b* = −0.01, *β* = −0.15, *SE* = 0.004, *p* = 0.05, but not to donate, *b* = −0.03, *β* = 0.12, *SE* = 0.02, *p* = 0.14, or intend to purchase green products, *b* = 0.01, *β* = 0.07, *SE* = 0.01, *p* = 0.39.

Next, we examined whether the priming manipulation had the desired effects on state optimism. Results showed that reading the optimistic climate article (versus the pessimistic article) positively predicted subsequent state optimism, *b* = 0.42, *β* = 0.24, *SE* = 0.14, *p* = 0.004. In turn, state optimism positively predicted green purchasing intent, *b* = 0.27, *β* = 0.30, *SE* = 07, *p* < 0.0001, donating one’s earnings from the study to the WWF, *b* = 0.06, *β* = 0.16, *SE* = 0.03, *p* = 0.05, support for geoengineering, *b* = 0.24, *β* = 0.32, *SE* = 0.06 *p* < 0.001, and modestly predicted joining the environmental activism group, *b* = 0.08, *β* = 0.14, *SE* = 0.08, *p* = 0.09. Examination of the indirect effects showed that induced state optimism significantly mediated the links between the priming task and green purchasing intent, *b* = 0.11, *β* = 0.07, *SE* = 0.05, *p* = 0.004, LLCI = 0.03 ULCI = 0.25, donating, *b* = 0.02, *β* = 0.04, *SE* = 0.01, *p* = 0.008, LLCI = 0.01 ULCI = 0.06, geoengineering, *b* = 0.10, *β* = 0.08, *SE* = 0.05, *p* = 0.004, LLCI = 0.03 ULCI = 0.20, and joining the environmental group, *b* = 0.03, *β* = 0.03, *SE* = 0.02, *p* = 0.042, LLCI = 0.008 ULCI = 0.25. The model fit the data well, *χ*^2^ = 4.71 (*df* = 9, *p* = 0.52), RMSEA = 0.00 (95% CI = 0.00–0.05), CFI = 1.00, NFI = 0.96.

## Discussion

Optimism can imbue people with hope, self-efficacy, and the capacity to persist and achieve goals when faced with challenges and uncertainty (Carver et al., 2010). Optimists are also approach-oriented and possess a greater capacity to tackle stressful life events (Nes and Segerstrom, 2006). Given that environmental dilemmas are complex and difficult to predict (discussed in Davis and Stroink, 2016), and sometimes evoke feelings of anxiety and stress (Clayton, 2020), optimism might encourage taking action to benefit the environment. Optimism is also entwined with positive emotionality and life satisfaction (i.e., subjective wellbeing), which seems to promote, and be a consequence of, engaging in pro-environmental behavior (PEB; see Zawadzki et al., 2020 for meta-analysis). Nonetheless, evidence has been mixed regarding whether trait optimism or closely related constructs, such as hope and future subjective wellbeing, are reliably associated with greater PEB (Ojala, 2012, 2015; Kaida and Kaida, 2016a,b, 2017). Similar mixed findings have been reported for the relations among support for geoengineering technologies, optimism, and engagement in conservation behavior (Murtagh et al., 2015; Rehman et al., 2021). Most of the research on the associations between subjective wellbeing, optimism–pessimism, and PEB has been cross-sectional, precluding an examination of “true” causal mechanisms. This is particularly important given some evidence that, like subjective wellbeing, optimism might be an antecedent and/or an outcome of PEB (Kaida and Kaida, 2019). Moreover, trait-level as opposed to state-level optimism–pessimism has received more attention in the literature, and more research involving an assessment of optimism–pessimism specifically toward environmental dilemmas is needed (Morris et al., 2020).

To attend to these gaps, we tested whether priming environmentally related optimism (versus pessimism) would increase state optimism (Hypothesis 1) to consequently predict intentions to purchase “green” products, as well as willingness to donate to the World Wildlife Fund (WWF) and join an environmental organization (Hypothesis 2). Controlling for participant sex and age, in line with some previous research (e.g., Kaida and Kaida, 2017, 2019), we found support for the idea that priming people to feel hopeful about the future of the environment heightened their optimism, which promoted intentions to make green purchases and donate to the WWF. This is in contrast to findings by Morris et al. (2020), where pessimistic affective messaging revolving around beekeeping and climate change increased risk perceptions and greater outcome efficacy (believing that one had the power to influence climate change). However, neither retrospective nor actual PEB were examined in their study.

Some have posited a reliance on science and technology to solve environmental ills is problematic and may breed complacency and inaction regarding conservation behavior (Dunlap et al., 2000; Murtagh et al., 2015; Mittiga, 2019). Nonetheless, support for geoengineering technologies to abate climate change has been linked with expressing concern for the environment (Landry et al., 2018), and people seem to express optimism toward to efficacy of geoengineering interventions to mitigate environmental dilemmas (Rehman et al., 2021). We found that optimistic environmental messaging promoted support for geoengineering *via* heightened state optimism (Hypothesis 2).

### Limitations

The current study has several noteworthy strengths, such as the use of an experimental design with *in vivo* assessments of PEB (e.g., donation behavior). Nonetheless, there are important limitations to consider. The *in vivo* measures of donating behavior and committing to join a naturalist organization were uncorrelated with one another, which may signal issues with convergent validity. MTurk workers could also be much less inclined to donate their study earnings in comparison to other community-level populations and undergraduate students, and perhaps this is a problematic means of assessing *in vivo* PEB in this population. Furthermore, evidence suggests that optimism may be both an antecedent and an outcome of PEB (Kaida and Kaida, 2019); however, we were unable to assess the latter causal pathway (i.e., PEB ➔ state optimism). Therefore, in future experimental work, it would be fruitful for researchers to examine whether *in vivo* PEB can also enhance state optimism. Moreover, we decided to focus on state optimism–pessimism to address important causal issues lacking in extant research, yet it may be prudent to control for trait-level dispositions to ascertain the unique contributions of state-level influences more confidently (see Kluemper et al., 2009). Future researchers might consider the potential moderating role of environmental concern to the relationship between optimism induction and PEB. Rafiq et al. (2022) recently found that environmental concern moderated the relationship between dispositional optimism and eco-friendly tourist behavior, such that those high in concern and high in optimism were most eco-friendly in their tourism actions. Perhaps trait environmental concern would similarly interact with induced state optimism in predicting a wider range of pro-environmental actions. It is also possible that optimism priming could induce group differences in environmental concern, which might then mediate links between optimism priming and PEB. Finally, our experimental design relied on examining between-group differences. It would also be interesting to expose participants to both optimistic and pessimistic messaging using a within-subject design to better target intra-individual changes in the effects of optimism and pessimism on environmentalism.

## Conclusion

Gathering insight into the downstream influences of optimistic and pessimistic environmental messaging on actual green behavior and support for techno-centric solutions to environmental dilemmas, such as geoengineering, is paramount (Morris et al., 2020). Our findings suggest that optimistic environmental messaging heightens one’s sense of optimism temporarily, which then promotes behaving in an ecologically conscious way, as well as being aware and supportive of climate abating technologies. These results coincide with some research (e.g., Kaida and Kaida, 2017), but contrast others (e.g., Morris et al., 2020). The current study attends to several shortcomings of previous work in that it was an experiment where both state optimism and *in vivo* PEB were assessed to provide a more valid test of causality. Our findings indicate that optimistic messaging enhanced state optimism and behaving in ways that will benefit the environment. However, our findings also highlight a problem raised by others, in that optimistic environmental messaging has been declining since the early 1970s (McAfee et al., 2019). Together, these findings highlight a troubling contradiction: Although climate change is a progressively worsening global issue that must be covered diligently and accurately in the popular press, it is simultaneously important to increase focus and coverage on positive steps being taken toward bettering our climate. Indeed, others have highlighted the fact that some meaningful advancements in habitat and species conservation and rehabilitation, reducing commercial fishing impacts, and the scope of legislative environmental protection have been made (McAfee et al., 2019) alongside a global greening phenomenon (Piao et al., 2020), and yet receive little relative media attention (Ridley, 2020). Ultimately, our findings suggest that this could negatively impact individuals’ environmental engagement.

## Data Availability Statement

The dataset presented in this study can be found in an online repository on the Open Science Framework: https://osf.io/gqv94/.

## Ethics Statement

The study involving human participants was reviewed and approved by the Nipissing University Research Ethics Board. The participants provided their written informed consent to participate in this study.

## Author Contributions

MM collected the data, performed data analysis, and helped to prepare the manuscript. AD co-wrote the manuscript. SA designed the study and led the revision of the manuscript. All authors contributed to the article and approved the submitted version.

## Funding

Funding for this research provided by a Social Sciences and Humanities Research Council of Canada (SSHRC) Institutional Research Grant (file # 102093) awarded to SA.

## Conflict of Interest

The authors declare that the research was conducted in the absence of any commercial or financial relationships that could be construed as a potential conflict of interest.

## Publisher’s Note

All claims expressed in this article are solely those of the authors and do not necessarily represent those of their affiliated organizations, or those of the publisher, the editors and the reviewers. Any product that may be evaluated in this article, or claim that may be made by its manufacturer, is not guaranteed or endorsed by the publisher.
