# Supplementary material for: Optimistic Environmental Messaging Increases State Optimism and in vivo Pro-environmental Behavior
Source: Front Psychol. 2022 Apr 29;13:856063. doi: 10.3389/fpsyg.2022.856063 (PMC9103192; doi:10.3389/fpsyg.2022.856063)
Supplement: Supplementary file 1 [file Data_Sheet_1.pdf]

frontiers

Supplementary Material

1 Bogus Environmental Optimism Article

Taken and adapted from:

Coster Content (2019, March 8). *Three big reasons to be optimistic about our environment in 2019*. Coster Content. https://www.costercontent.co.uk/blog/optimism-environment

Pundy, D. (2019, August 2). *Five reasons to be optimistic about the environment*. E- mc^2 Energy Matters to Climate Change. https://www.e-mc2.gr/el/news/five-reasons-be-optimistic-about-environment

Steffen, A. D. (2019, March 21). *Coal and gas are losing the economic battle to renewables*. Intelligent Living. https://www.intelligentliving.co/coal-gas-losing-economic-battle-renewables/

Vaughn, A. (2019, February 25). *Renewable energy will be world's main power source by 2040, says BP*. Euractiv. https://www.euractiv.com/section/energy-environment/news/renewable-energy-will-be-worlds-main-power-source-by-2040-says-bp/

POSITIVITY FOR OUR PLANET

REASONS TO BE OPTIMISTIC ABOUT OUR ENVIRONMENT IN 2021

BY: JONAH WELLS **DECEMBER 8, 2020**

Optimism is key for driving positive change.

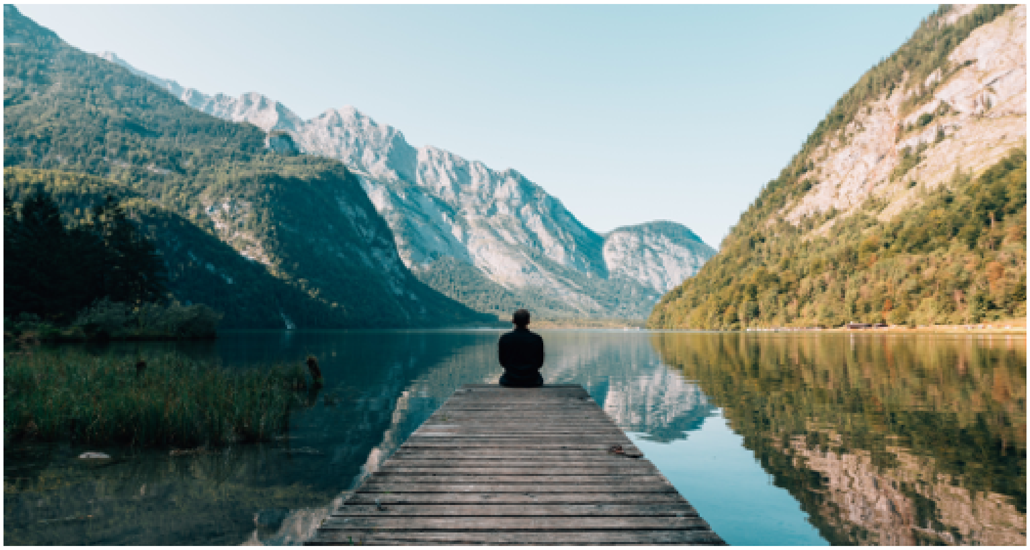

Let's take the time to reflect on the changes we are making towards a greener world. Optimism is a driving force and emphasis has to be put on the progress we've made in the last decade to further drive positive change.
Here are four big examples of the positive changes being made to support a happier, healthier planet.

Conservation Efforts are Saving Many Endangered Species

For instance, Germany, where wolves were extinct for 150 years, is now home to several dozens of wolf packs. And to some of them, Germany has become too small. Similar trends are being seen in North America, and other species, like the beaver and the European bison are on the rise again in Europe and are being spotted in the wild more often.

Renewable Energy Use is Climbing Fast Along with its Technology

Every city or country that is committed to a complete transition to renewable energy is adding to the global momentum towards a zero-carbon future.
There are no doubts that this momentum is speeding up – over 100 cities and countries are now committed to being powered on 100% clean energy.
The good news doesn't stop there – Portugal ran on renewable energy every day in March of 2020, and Germany produced enough renewable energy in the first 6 months of 2018 to power every household for a year.
Battery technology is coming along in leaps and bounds alongside solar panel efficiency – both of which pave a new path for the future of energy.
According to Bloomberg New Energy Finance (BNEF), renewables are now the cheapest form of new electricity generation across two thirds of the globe. By 2030, wind and solar power will "undercut existing coal and gas almost everywhere," according to BNEF's New Energy Outlook 2019.

24

Even the oil majors now acknowledge that renewables are the future. This year, the UK-based oil company BP said wind, solar and other renewables will account for about 30% of the world's electricity supplies by 2040, up from 25% in its previous estimates.
And the speed of growth was without parallel, the company said in its annual energy outlook. In fact, most European oil majors have started diversifying their assets and are now pouring billions into clean technologies.

The Divestment Movement Grows from Strength to Strength
Grassroots student-led divestment movements have swept over universities all over the world. Their aims are simple: to highlight awareness surrounding the dangers of climate change and put a stop to investment in fossil fuels and non-renewable energy sources.
In the UK, it has been tremendously successful. At least 76 universities have committed to divestment, which equals to around £12 billion in investment that will not fuel some of the world's most profitable oil companies in the world - such as ExxonMobil, Shell,

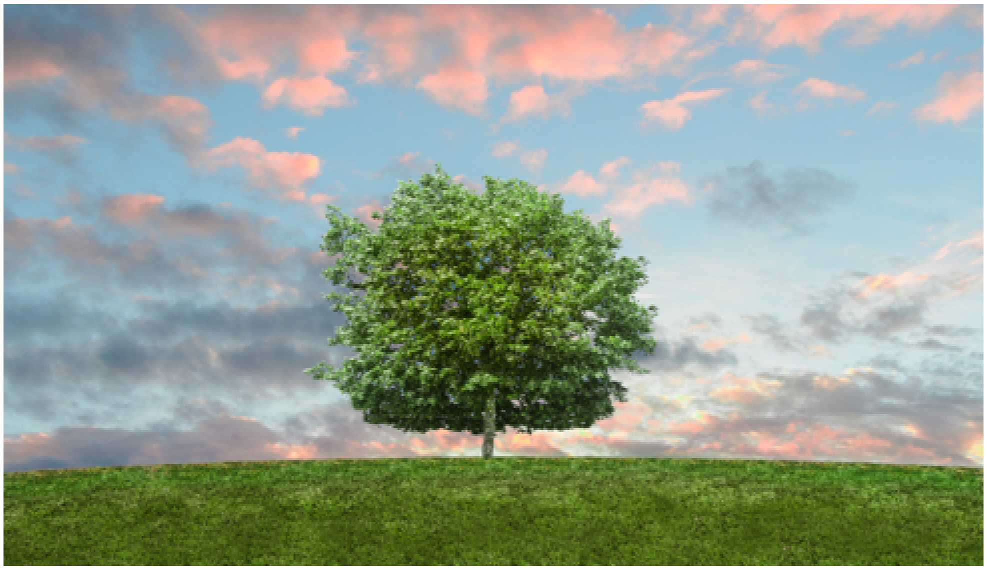

Divestment has spread far beyond universities and continues to gather momentum – now Religious institutions, pension funds, and private companies are beginning to realise non-renewable energy sources will inevitably make way for cleaner and greener sources of energy.

The Next Generation is Taking to the Street
Climate and environmental rights marches have been happening around the world for decades. But the 24 hours of climate action on March 15th, 2019 felt different – there were over 2,000 protests in 125 countries, with more than 1 million students skipping school to participate.

The anger, fear, and disappointment of a younger generation has turned into inspiring action full of hope that demands a greener world. The magnitude of these calls continues to pile on the pressure for major changes in environmental policy across the world.

Every country, community, and individual has the choice to use positive progress as a driving force for an even better world. Optimism isn't always easy when it feels like we're surrounded by bad news. But regularly reminding ourselves how far we've come can help inspire action and change the tide towards a sustainable future.

WE'RE CELEBRATING TEN YEARS OF AMAZING IMPACT - SUPPORT OUR NEXT TEN YEARS

25

Supplementary Figure 1. Bogus environmental optimism article.

2 Bogus Environmental Pessimism Article

Taken and adapted from:

Wallace-Wells, D. (2017, July 9). *The uninhabitable Earth famine, economic collapse, a sun that cooks us: What climate change could wreak — sooner than you think*. New York Magazine. https://nymag.com/intelligencer/2017/07/climate-change-earth-too-hot-for-humans.html

NO HOPE FOR OUR ENVIRONMENT

THE WINDOW FOR ACTION HAS CLOSED

BY: JONAH WELLS DECEMBER 8, 2020

It is, I promise, worse than you think.

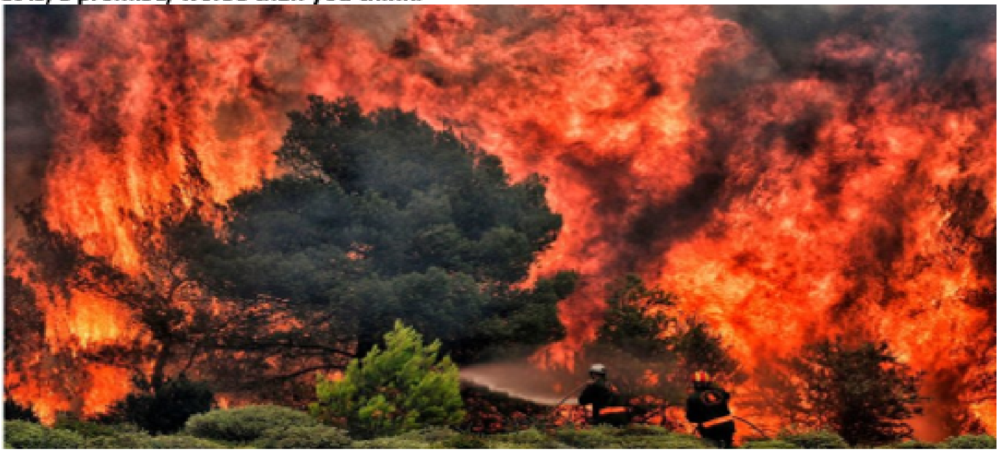

It is, I promise, worse than you think. In the near future, large parts of the Earth will likely become close to uninhabitable, and other parts horrifically inhospitable, as soon as the end of this century.

Even when we train our eyes on climate change, we are unable to comprehend its scope.— the destruction we've already baked into our future — is horrifying enough. Most people talk as if Miami and Bangladesh still have a chance of surviving; most scientists assume we'll lose them within the century, even if we stop burning fossil fuel in the next decade.

If the planet is five degrees warmer at the end of the century, we will have as many as 50 percent more people to feed and 50 percent less grain to give them. Drought might be an even bigger problem than heat, with some of the world's most arable land turning quickly to desert. By 2080, much of the world will be in permanent extreme drought. None of these places, which today supply much of the world's food, will be reliable sources of any.

Global ecosystem destruction will bring about an increase disease. You don't worry much about dengue or malaria if you are living in New York or Toronto. But as the tropics creep northward and mosquitoes migrate with them, you will.

Other stuff in the hotter air is even scarier, with small increases in pollution capable of shortening life spans by ten years. The warmer the planet gets, the more ozone forms, and by mid-century, Americans will likely suffer a 70 percent increase in unhealthy ozone smog, the National Center for Atmospheric Research has projected. By 2090, as many as 2 billion people globally will be breathing air above the WHO "safe" level.

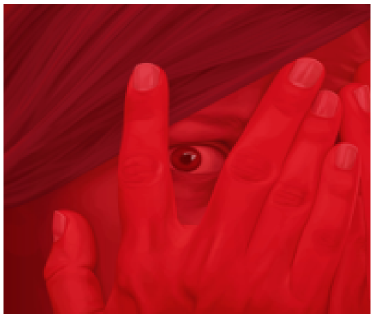

24

Overall, social conflict could more than double this century. This is one reason that the U.S. military is obsessed with climate change: What accounts for the relationship between climate and conflict? Some of it comes down to agriculture and economics; a lot has to do with forced migration, already at a record high, with at least 65 million displaced people wandering the planet right now.

Every degree Celsius of warming costs, on average, 1.2 percent of GDP (an enormous number, considering we count growth in the low single digits as "strong"). This is the sterling work in the field, and their median projection is for a 23 percent loss in per capita earning globally by the end of this century (resulting from changes in agriculture, crime, storms, energy, mortality, and labor).

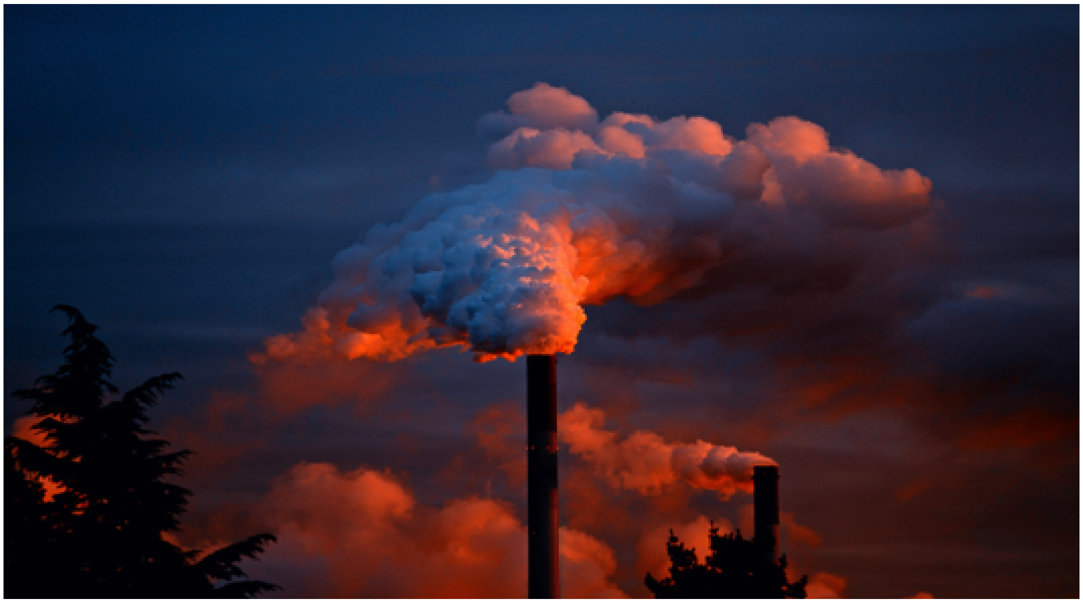

Everyday 'green' behaviors available to the average citizen are nearly inconsequential. For example, over 80% of the plastics that we put in our recycling bins each week are not actually recyclable – they will wind up in a landfill, burned, or in the ocean. Those that are can be recycled once or twice before the plastic is no longer recyclable. Most of your carbon footprint is taken up by things you cannot control in society – road construction, manufacturing, and other factors that are passed on to the individual, whereas nearly all pollution is created by large industries that are protected by legislation that allows them to continue to pollute, and by a capitalist market that creates never-ending demand for their products.

Yet, even if we were to stop burning fossil fuels today, it would still be too late. We have passed the tipping point for polar ice loss and ocean temperature rise that has created a cycle of unstoppable global temperature rise.

Soon, the Earth's ecosystem will boil with so many natural disasters that we will just start calling them "weather": a constant swarm of out-of-control typhoons and tornadoes and floods and droughts, the planet assaulted regularly with climate events that not so long ago destroyed whole civilizations. And at this point, our window for action has closed; there is nothing we can do to stop it.

WE'RE CELEBRATING TEN YEARS OF AMAZING IMPACT - SUPPORT OUR NEXT TEN YEARS

25

Supplementary Figure 2. Bogus environmental pessimism article.

3 Wording of Questions for In-Vivo Pro-Environmental Behavior

3.1 Script: Donating to World Wildlife Fund

Instructions: "*We are offering the opportunity for you to donate your prize money to the World Wildlife Foundation (WWF). Please indicate whether you would like to keep the money you earned from this study or gave your earnings donated anonymously to WWF.*"

3.2 Script: Joining Bogus Environmental Group

Instructions "*There is a new Environmental Awareness group called North American Environmentalists (NAE). Members attend virtual meetings to discuss environmental issues that are important to North America, attend political demonstrations relevant to environmental protection, and engage in activities such as community cleanups. If you would like to join, please provide your name and email address here (this page will be removed from the rest of your anonymous survey).*"
